# Microbial Metabolite 3-Indolepropionic Acid Mediates Immunosuppression

**DOI:** 10.3390/metabo12070645

**Published:** 2022-07-14

**Authors:** Carlos Guijas, Lucy E. Horton, Linh Hoang, Xavier Domingo-Almenara, Elizabeth M. Billings, Brian C. Ware, Brian Sullivan, Gary Siuzdak

**Affiliations:** 1 Scripps Center for Metabolomics, The Scripps Research Institute, 10550 North Torrey Pines Road, La Jolla, CA 92037, USA; carlos.guijas@gmail.com (C.G.); lhoang@scripps.edu (L.H.); ebilli@scripps.edu (E.M.B.); 2 Department of Immunology and Microbiology, The Scripps Research Institute, 10550 North Torrey Pines Road, La Jolla, CA 92037, USA; lhorton@scripps.edu (L.E.H.); bware@scripps.edu (B.C.W.); 3 Computational Metabolomics for Systems Biology Lab, Omics Sciences Unit, Eurecat—Technology Centre of Catalonia, 08005 Barcelona, Catalonia, Spain; xavier.domingoa@eurecat.org; 4 Departments of Chemistry, Molecular, and Computational Biology, The Scripps Research Institute, 10550 North Torrey Pines Road, La Jolla, CA 92037, USA

**Keywords:** activity metabolomics, cytotoxic T lymphocytes, T-cell exhaustion, immunosuppression, lymphocytic choriomeningitis virus

## Abstract

The microbial-derived metabolite, 3-indolepropionic acid (3-IPA), has been intensely studied since its origins were discovered in 2009; however, 3-IPA’s role in immunosuppression has had limited attention. Untargeted metabolomic analyses of T-cell exhaustion and immunosuppression, represented by dysfunctional under-responsive CD8^+^ T cells, reveal a potential role of 3-IPA in these responses. T-cell exhaustion was examined via infection of two genetically related mouse strains, DBA/1J and DBA/2J, with lymphocytic choriomeningitis virus (LCMV) Clone 13 (Cl13). The different mouse strains produced disparate outcomes driven by their T-cell responses. Infected DBA/2J presented with exhausted T cells and persistent infection, and DBA/1J mice died one week after infection from cytotoxic T lymphocytes (CTLs)-mediated pulmonary failure. Metabolomics revealed over 70 metabolites were altered between the DBA/1J and DBA/2J models over the course of the infection, most of them in mice with a fatal outcome. Cognitive-driven prioritization combined with statistical significance and fold change were used to prioritize the metabolites. 3-IPA, a tryptophan-derived metabolite, was identified as a high-priority candidate for testing. To test its activity 3-IPA was added to the drinking water of the mouse models during LCMV Cl13 infection, with the results showing that 3-IPA allowed the mice to survive longer. This negative immune-modulation effect might be of interest for the modulation of CTL responses in events such as autoimmune diseases, type I diabetes or even COVID-19. Moreover, 3-IPA’s bacterial origin raises the possibility of targeting the microbiome to enhance CTL responses in diseases such as cancer and chronic infection.

## 1. Introduction

The metabolite, 3-indolepropionic acid (3-IPA), was discovered to be exclusively derived from gut microbiota in 2009 [1] and has since been extensively studied. 3-IPA is biosynthetically derived from tryptophan by *Clostridium sporogenes* and shown to have immunomodulatory properties [1,2]. However, its role in immunosuppression has received limited attention; this is especially surprising because of 3-IPA’s microbial origins and the need for gut microbiota to evade the immune response. To examine immunosuppression in general and to specifically determine if 3-IPA plays a role, we created a set of unbiased T-cell exhaustion experiments using untargeted metabolomics with the aim of identifying key metabolites associated with this phenomenon.

T-cell exhaustion represents a dysfunctional state of antigen-specific T cells in response to certain viral, bacterial and parasitic persistent infections as well as during cancer pathogenesis [3,4]; thus, it is a state that while initiated by a particular vector/pathology, it is yet promulgated by the immune system. This phenomenon was discovered and characterized through the in vivo infection of C57BL/6 mice (B mice) with the lymphocytic choriomeningitis virus (LCMV) Clone 13 (Cl13) [5]. Unlike similar strains, including LCMV Armstrong infection that results in a robust T-cell response capable of completely clearing the virus, LCMV Cl13 produces a chronic infection. Although the virus can persist in the host organism indefinitely, the Cl13 variant does not shorten the lifespan of normal mice. This persistent infection is mainly mediated by an immunosuppressed CD8^+^ T-cell (CTL, cytotoxic T lymphocytes) responses (exhaustion) [3,4]. There is a gradual loss-of-function of CTL following LCMV Cl13 infection that includes key responses such as cytokine release, proliferative capacity and cytotoxic activity. For this reason, LCMV CL13 infection of certain strains of mice has been extensively used for the characterization of T-cell exhaustion, allowing for the discovery of several signaling pathways and cytokines involved in these responses. This knowledge has formed the basis for checkpoint therapy against cancer [6,7] as well as proving useful in promoting negative immune responses in diseases such as sepsis, autoimmune diseases or organ transplantation [8,9].

Recently, the Sullivan lab has reported that certain strains of mice, including FVB/N (F mice), NZO and NZB mice, develop a lethal infection with LCMV Cl13 that is mediated by the robust activation of CTL [4,10]. CTL over-activation results in mice death 7–9 days post-infection due to the fact of pulmonary failure. This fatal outcome is a result of leakage of pulmonary exudate into the lungs due to the increase in pulmonary endothelial vascular permeability. Death can be prevented by deleting circulatory CD8^+^ cells or blocking type-I interferon, which is essential for the generation of a hyperfunctional CTL response [4,10] early after infection. This research provides an excellent framework for the discovery and characterization of events that may be mediating mice death to LCMV Cl13 infection through comparison with the exhausted CTL-mediated responses in the strains of mice that survived with a persistent infection. Similarly, novel negative CTL regulators can be identified. This new approach switches the paradigm for the discovery of immunosuppressant cytokines and pathways. Instead of using two different types of infection routes (i.e., LCMV Armstrong versus LCMV Cl13), this instead uses LCMV C113 solely in different mice strains, thus allowing for a phenotypic readout survival with a persistent infection due to the CTL negative immune regulation (e.g., B mice) or death due to the acute CTL responses (e.g., F mice).

Many negative immune regulators have been discovered and/or characterized through the study of CTL exhaustion. These include programmed death-1 (PD-1) and its ligand (PD-L1), interleukin 10 (Il10), cytotoxic T lymphocyte antigen-4 (CTLA-4), T-cell membrane protein-3 (TIM-3), lymphocyte-activation gene 3 (LAG3) and tumor necrosis factor alpha-induced protein 8-like 2 (TIPE2), among others [3,6,8,11,12,13]. Although the role of endogenous metabolites in regulating these responses has been under-investigated for decades, recently, several metabolites with immunomodulatory properties have been found beyond the traditional oxylipins, arachidonic acid and omega-3 fatty acids [14,15] that are associated with immunomodulation. For example, in the last three years, itaconate [16], alpha-ketoglutarate [17], cis-7-hexadecenoic acid [18], L-arginine [19] and tetrahydrobiopterin [20], among others, have been demonstrated to play pivotal roles in immune responses through the modulation of different immune cell types. This blooming has been achieved, in part, through the development of new metabolomics technologies [21,22]. The close proximity between phenotypic and metabolic changes makes metabolomics a fascinating technology to screen for the metabolic changes linked to different phenotypic outcomes to prioritize metabolites for activity testing. For the latter, the incorporation of pathway analysis and artificial intelligence has been demonstrated as an effective approach for predicting active metabolites [23,24,25,26]. The recent discovery of certain mice strains that respond to LCMV Cl13 infection with a robust CTL response that result in death provides a solid model for the characterization of endogenous metabolites that can be mediating these systemic responses.

Here, we compared two highly genetically related mice, DBA/1J and DBA/2J, to study the systemic metabolic response to in vivo infection with LCMV Cl13. Unsurprisingly, one of the highest prioritized metabolites in these studies was the microbial-derived metabolite, 3-IPA. Further investigation found that 3-IPA plays a pivotal role in the negative immune regulation of several CTL responses during LCMV Cl13 infection, affecting mice survival. Supplementation with 3-IPA in the drinking water allows certain strains of mice to survive longer to deadly LCMV Cl13 infection. Moreover, the discovery of this negative immunoregulatory metabolite raises the possibility to target the microbiome for the modulation of the CTL-mediated immune homeostasis in cancer, autoimmune diseases, sepsis or organ transplantation.

## 2. Results and Discussion

### 2.1. Global Metabolomics Shows Differential Responses of Mice to LCMV Cl13 Infection

**Figure 1:**
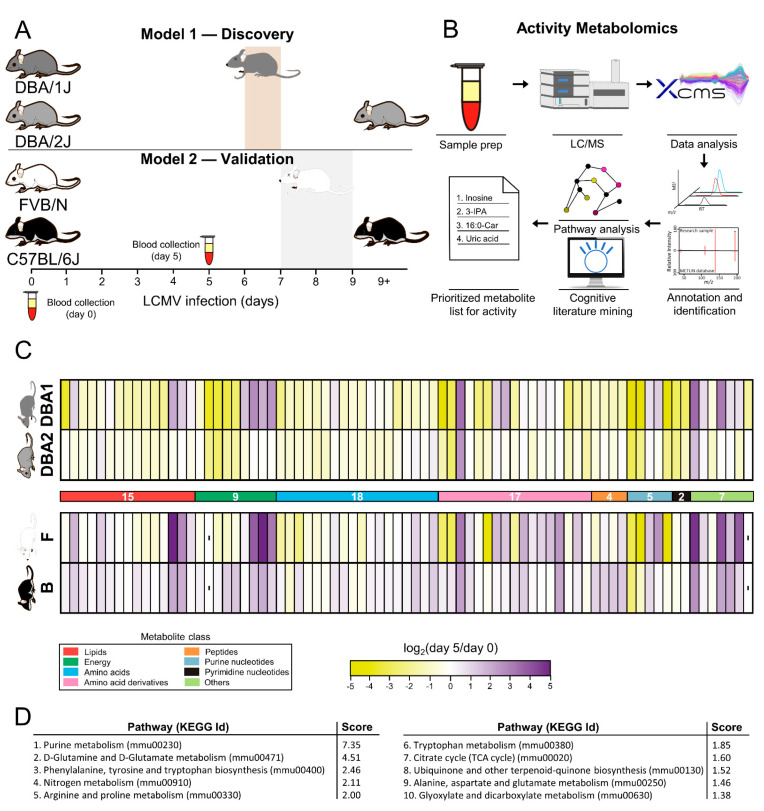
Untargeted metabolomic experiments were designed to identify dysregulated metabolites for later testing for activity. (**A**) Plasma was collected prior to virus inoculation (day 0) and after 5 days of infection, 24–48 h prior to DBA1 mice death. For validation, a similar analysis was performed separately on C57BL/6J (B mice) and FVB/N (F mice), a model that has shown similar behavior in the response to LCMV Cl13. The known phenotypic outcomes in both models enhanced our chance of observing relevant metabolites associated with CTL responses and survival. (**B**) Untargeted metabolomics pathway analysis and cognitive-driven accelerated literature mining were used to prioritize those metabolites that might play significant roles in mediating the differential T-cell responses. (**C**) Over 70 metabolites were found to be dysregulated in the DBA model between day 0 and 5 of LCMV Cl13 infection (*q* < 0.05, one-way ANOVA followed by a Kruskal–Wallis nonparametric test). All but two metabolites (citric acid and N-acetylmuramic acid) were also detected in the validation model. Metabolite changes at day 5 were calculated using day 0 levels as a reference and represented in a heatmap using a logarithmic scale. (**D**) Pathway analysis at day 5 after inoculation was carried out to predict the metabolic pathways that were more dysregulated between mice that survived and died.

Global metabolomics is an evolving technology for the discovery of dysregulated metabolites that modulate physiology and is typically employed by performing comparisons between similar biological systems with different phenotypic outcomes [21,22]. In these experiments, we compared LCMV Cl13-infected mouse strains that died due to the hyperactivation of CTLs to infected mice strains that survived with persistent infection as a result of T-cell exhaustion [4] to identify metabolites that are associated with different immune responses to LCMV Cl13 (Figure 1a). This was initially performed through the analysis of plasma from FVB/N (F) mice (died 7–9 days after LCMV Cl13 inoculation) and C57BL/6J (B) mice (survived with a persistent infection), showing that a great number of metabolic features were altered prior to mice inoculation with the virus at day 0 (Table 1), hindering the untargeted analysis. However, the genetic variability between infected mouse strains that resulted in a persistent infection and those that resulted in a lethal infection made comparative analysis challenging, since basal (unstimulated) changes were expected to be high.

To compensate for this challenge, a different mouse model with a more closely related background and likely a more similar basal metabolome was tested for these analyses. It was discovered that DBA/1J (DBA1) and DBA/2J (DBA2), two closely related strains of mice that differ only by 5.6% at the single-nucleotide polymorphism (SNP) level [27,28], responded differentially to LCMV Cl13 infection. Where DBA2 mice showed immunosuppressed T-cell responses that resulted in a persistent infection that did not affect normal lifespan (similar to C57BL/6J), DBA1 mice showed infection that was followed by a robust T-cell response resulting in death typically 6 days after inoculation (similar to FVB/N) (Figure 1a). DBA1 and DBA2 mice strains originate from a common source, the oldest inbred strain DBA [29]. Despite their common origin, DBA1 and DBA2 differ at several loci related with immune response. For this reason, these models have been employed for the study of several immune-based diseases such as rheumatoid arthritis [30], atherosclerosis [31] as well as neurobiological processes [32,33]. The common origin of both mice strains, together with the different response to LCMV Cl13, made this model an interesting framework for the study of metabolic modulators of T-cell responses and, ultimately, survival. The basal metabolome similarity of these strains was higher than in the other model, as it was demonstrated by the analysis of changed metabolic features in plasma of animals at day 0, prior to infection (Table 1).

Untargeted metabolomics profiling was performed on plasma samples of LCMV Cl13-infected DBA1 and DBA2 mice. Plasma was used to investigate systemic changes upon infection, since it is composed of the end products of the global metabolism of tissues and organs and, therefore, reflects tissue-specific metabolism [34]. Plasma was collected prior to virus inoculation (day 0) and after 5 days of infection, 24–48 h prior to DBA1 mice death. To validate the metabolites found in this model, a similar analysis was performed separately on B and F mice, a model that has shown a similar behavior in the response to LCMV Cl13 (Figure 1A). Untargeted metabolomics raw results were subjected to feature annotation and metabolite identification by comparison with curated MS/MS data [35,36,37]. Pathway analysis and cognitive-driven accelerated literature mining were used to prioritize those metabolites that might play significant roles in mediating the differential T-cell responses to Cl13 and, subsequently, mice survival (Figure 1B).

Over 70 metabolites were found to be dysregulated in the DBA model (one-way ANOVA followed by a Kruskal–Wallis nonparametric test). All but two (citric acid and N-acetylmuramic acid) were also detected in the validation model. If metabolites are divided by biochemical class, lipids, amino acids and their derivatives represent 65% of the total metabolites observed to be dysregulated (Figure 1C). The logarithmic fold change of the metabolites between day 0 and day 5 of infection with Cl13 were represented in heatmaps, and metabolic changes in deadly infections were greater than in persistent infections for most of metabolites (65/77 for the DBA model and 51/75 for the validation model). This is especially relevant for energy metabolites (acylcarnitines and central carbon metabolites). Even though the response of this group of metabolites to the Cl13 variant was not the same (approximately half of the metabolites increased and half decreased in both models), changes were greatly reduced in mice that survived compared to those that cannot overcome the infection. This observation was similar for some amino acid derivatives and purine nucleotides. All metabolites integration areas and standard deviations are available in the Supplementary Materials Table S1. Raw data were deposited in the XCMS Online Public Repository.

To extract global information from the metabolic changes measured, a pathway analysis at day 5 after inoculation was carried out to predict the metabolic pathways that were more dysregulated between mice that survived and died. Purine metabolism was predicted to be the most dysregulated pathway by far, in accordance with the important number of purines found dysregulated and their differential behavior in both models. Due to the high number of amino acids and amino acid derivatives found, many amino acids metabolic pathways were predicted. Among those, we found glutamine, glutamate, phenylalanine, tyrosine, arginine and tryptophan. The TCA cycle was also anticipated to be dysregulated during the mice response to Cl13 virus, explained by the detection of several energy metabolites related with that pathway (citric acid, aconitic acid, oxoglutaric acid and malic acid) (Figure 1D).

Altogether, we examined a model that allowed us to interrogate the metabolic changes that correlated LCMV Cl13 inoculation with differential immune responses resulting in mice death or survival. Overall metabolic remodeling was greater in mice whose CTL strongly responded to the virus inoculation, specifically being observed in purine nucleotides, energy pathways and amino acid metabolism.

### 2.2. 3-IPA and Purines Are Candidates to Modulate Response to LCMV CL13 Infection

**Figure 2:**
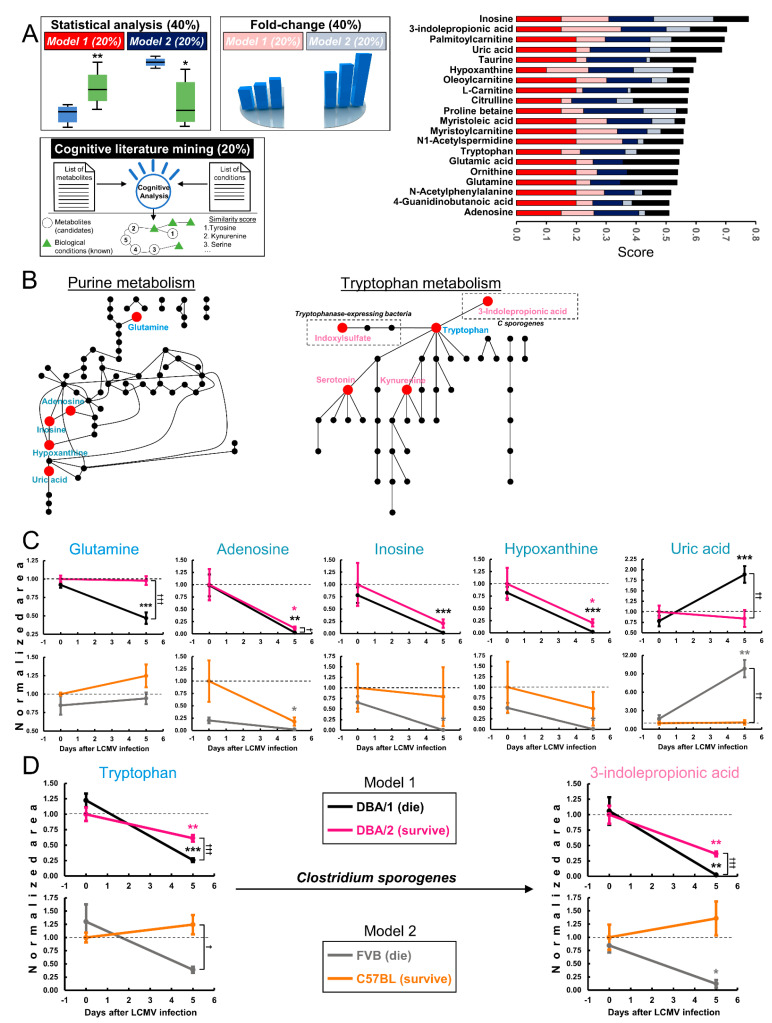
3-IPA and purines were prioritized as candidates to modulate immune responses to LCMV infection. (**A**) Metabolite candidates prioritized and chosen using a weighted algorithm employing statistical significance (40%), fold change (40%) and cognitive computing analysis (20%). The scoring (0 to 1) is represented on the color coded graph segmented into the weighed contributions of each factor. Red/dark blue represent statistics for the two mouse models, pink/light blue represent a fold change for the two mouse models, and black represents cognitive computing contributions. Full data can be found in the Supplementary Materials Table S2. (**B**) Dysregulated metabolites were projected (red dots) onto the purine and tryptophan metabolism pathways. (**C**) Purine metabolism intermediates fold change over the course of the LCMV infection for both models. (**D**) 3-IPA synthesis pathway metabolites fold change over the course of the LCMV infection for both models. Data were normalized using the strain that survived with a persistent infection (DBA2 and B mice) prior to the LCMV inoculation. Statistical comparisons were calculated in respect to the same animals at day 0 (* *q* < 0.05, ** *q* < 0.01, and *** *q* < 0.001, determined by one-way ANOVA followed by a Kruskal–Wallis nonparametric test) or between both animal colonies at day 5 (^†^ *q* < 0.05, ^††^ *q* < 0.01, and ^†††^ *q* < 0.001, determined by one-way ANOVA followed by a Kruskal–Wallis nonparametric test).

Metabolomics has been demonstrated as a powerful technology on its own for the discovery of metabolites that can be used as active drivers of biological processes. However, a prioritization step prior to the biological screening of metabolites is sometimes necessary, especially for in vivo models where testing all metabolites can require significant animal and time resources. For this reason, cognitive-driven accelerated literature mining has been recently demonstrated to be a useful artificial intelligence technology to identify those molecules that are closer to certain known phenotypes and rank them in terms of relationships found in literature [23,24,38]. In order to rank the over 70 metabolites found dysregulated by the untargeted metabolomics analyses, we used all the data and resources available including both mice models, statistical analysis of metabolites, fold changes and cognitive literature mining (Figure 2A and Supplementary Materials Table S2).

For the statistical and fold-change analyses, metabolites that had levels that were similar at day 0 but diverged upon 5 days of infection were prioritized, especially those that changed during the deadly infection. For the cognitive prioritization of metabolites, the similarity score given by the number of relationships in the literature between the provided known biological entities and the candidates to be ranked was used (Supplementary Materials Table S2A). The total score was an equally weighted composite score of the individuals scores generated by each mouse model (Figure 2a and Supplementary Materials Table S2). The colors in the composite score correspond to the model source for the score, and the size of each of these bars represents the share of the score from that model. Inosine was ranked as the first candidate to modulate immune responses to LCMV Cl13 infection, followed by 3-IPA, palmitoylcarnitine, uric acid and taurine (Figure 2A).

In addition to the purine pathway being the most dysregulated pathway at day 5 between mice that died and survived, all five purines measured were ranked among the top 20 candidates to be active metabolites. These endogenous molecules were glutamine and four of the terminal metabolites: adenosine, inosine, hypoxanthine and uric acid [39] (Figure 2B). Other more complex intermediary nucleotides in the pathway were not observed. Overall, it could be observed that terminal purines (i.e., adenosine, inosine and hypoxanthine) decreased over the course of the LCMV Cl13 infection for all mice. This decrease was especially dramatic in DBA1 mice. This response was qualitatively mimicked by F mice, although differences in purine nucleotide levels at day 0 were higher than in subsequent days (Figure 2C). The decrease in purines was reflected in the synthesis of uric acid in both deadly infected strains. Although some intermediates of its synthesis decreased upon 5 days of infection, uric acid levels remained unaltered for both mice strains that survived with a persistent infection (Figure 2C). In humans, uric acid is the terminal metabolite of the pathway, produced by the metabolism of purines by several organs resulting in its secretion into the bloodstream.

Conversely, in rodents, uric acid can be further metabolized into allantoin through three sequential reactions (Figure 2B). Allantoin could not be measured by a “semi-targeted” approach, supporting the idea that uric acid accumulation in the plasma of DBA1 and F mice was due to the accelerated metabolism of purines upon infection as well as the lack of further metabolization into allantoin (Figure 2C). On the other hand, glutamine was differentially regulated in the two DBA strains, but this behavior was not mimicked by the other mice model under investigation. Glutamine changes were challenging to relate with terminal purines, since there were many intermediate metabolites in the pathway and glutamine is also predicted to participate in many other biochemical routes. Purine metabolites have been demonstrated to be key regulators of immune function through several cell-type modulations including CTL [39]. Moreover, soluble uric acid has been described as an activator of the NLRP3 inflammasome [40]. These pieces of evidence, together with the different regulation of these pathways in mice that survived and died upon infection suggest that purine nucleotides could play a key role in CTL regulation and mice survival as was suggested by our prioritization ranking.

It was also observed that 3-IPA was ranked second (Figure 2A). This metabolite, discovered in 2009 by the Siuzdak laboratory as a microbiota-dependent catabolite of tryptophan in mice plasma, is exclusively synthesized by the gut commensal bacteria *Clostridium sporogenes* [1,2]. The tryptophan metabolism pathway was predicted to be dysregulated at day 5 of infection in the pathway analysis, and five of the metabolites of that pathway were found dysregulated by the untargeted analysis (Figure 2B). Curiously, indoxylsulfate, another tryptophan product dependent of microbiota catalysis was also changed in the untargeted analyses (Figure 2B). 3-IPA levels in fatal infections dropped to almost zero in both models. This decrease was milder in DBA2 mice, while levels remained steady in B mice (Figure 2D). Since the only reported precursor for the synthesis of 3-IPA is tryptophan, the levels of this amino acid followed a similar trend to 3-IPA in both models (Figure 2D). 3-IPA extinction upon deadly infection with LCMV Cl13 was similar to adenosine, inosine and hypoxanthine. The purines’ decrease can be explained in terms of uric acid accumulation, while the 3-IPA decrease might be explained by excretion or through the use of 3-IPA to modulate some of the responses that finally promote the fatal outcome.

### 2.3. 3-IPA Increased Mice Survival upon Fatal Infection with LCMV Cl13

**Figure 3:**
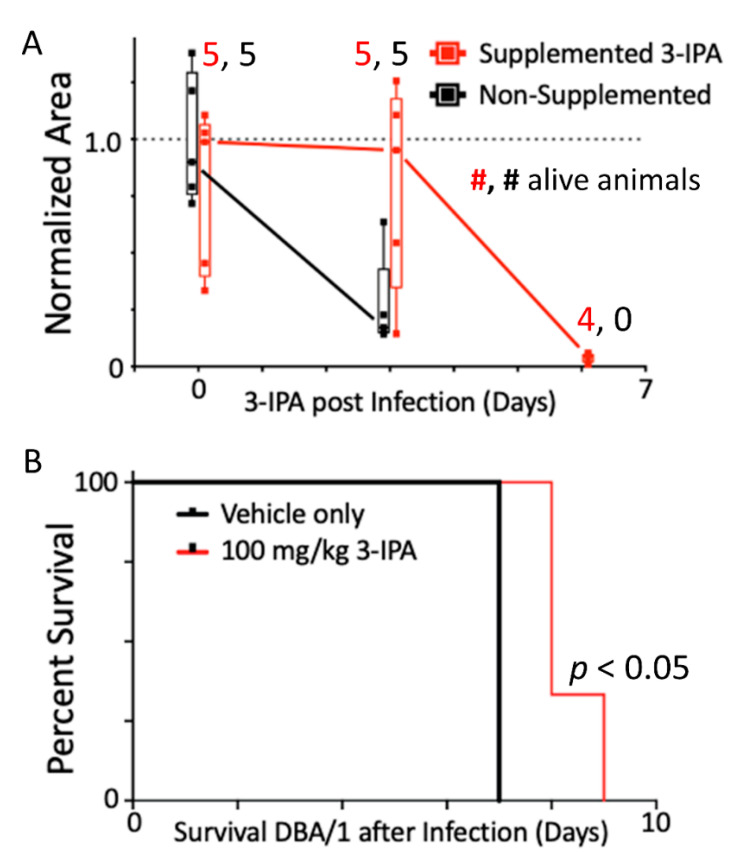
3-IPA supplementation helped in maintaining its plasma levels and delayed death in DBA1 mice infected with LCMV Cl13. (**A**) 3-IPA was measured in plasma post-LCMV Cl13 infection in supplemented (100 mg/Kg/day in the drinking water, 24 h prior to infection) and non-supplemented mice. Data were normalized using the non-supplemented cohort at day 0. The number of alive animals per group at each time point is indicated. (**B**) Survival of 3-IPA supplemented mice after LCMV Cl13 infection. Mice received either 100 mg/Kg/day of orally supplemented 3-IPA (*n* = 3) or vehicle alone (*n* = 4) daily from one day prior to infection. Drinking water for both groups contained powdered grape crush (2.3 g/L).

The high prioritization of 3-IPA led us to test supplementation of DBA/1J mice with 3-IPA was examined to determine survival outcomes. Because 3-IPA is produced by gut commensal bacteria [1], oral supplements of 3-IPA in the drinking water (100 mg/kg/day, starting 1 day before the virus inoculation) were provided. We observed significant increases in plasma 3-IPA levels in 3-IPA-treated mice at three days post-infection with levels decreasing to near zero by day 6 post-infection (Figure 3A). When evaluating survival for both groups, all 10 animals (*n* = 5 from each group) were alive at day 3 post-infection, although circulating 3-IPA was 75% lower in the non-supplemented cohort. At day 6 post-infection, the five animals of the control group had died, while four were still alive in the supplemented group. Nonetheless, the 3-IPA levels in this group were very low (95% lower than day 0 and day 3) but still measurable. Unsurprisingly, the only animal in the supplemented group that died at day 6 was the one with the lowest 3-IPA levels in plasma at days 0 and 3 (58% and 82% lower than the group average, respectively) (Figure 3A).

Since sedation and bleeding of mice at days 0 and 3 post-infection can affect mortality in LCMV Cl13-infected mice, we used a separate cohort of mice to assess survival. Similarly, a group of animals also received 3-IPA in the drinking water (100 mg/kg/day) from one day before the virus was inoculated. As expected, all non-supplemented animals died between day 6 and 7 post-infection. However, it was observed that mice receiving 3-IPA orally had a delayed death (days 8–9 post-infection) (Figure 3B).

Overall, we observed that DBA1 mice oral supplementation with 3-IPA helped maintaining its plasma levels during LCMV fatal infection, delaying mice death.

### 2.4. 3-IPA Suppressed Cytotoxic T-Lymphocytes Responses

**Figure 4:**
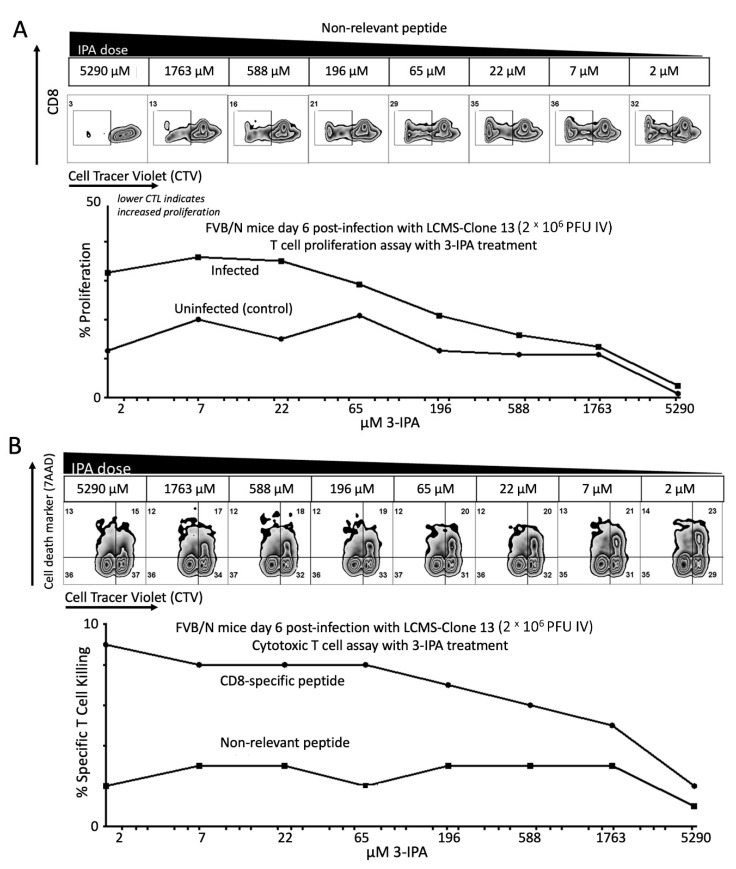
3-IPA suppresses cytotoxic T-lymphocytes responses. (**A**) 3-IPA dose-dependent suppression of CD8^+^ T-cell proliferation from infected FVB/N mice with LCMV Cl13 for 6 days. (**B**) 3-IPA dose-dependent suppression of target cell killing by CD8^+^ T cells from infected FVB/N mice with LCMV Cl13 for 6 days.

The LCMV Cl13 strain is commonly used to evaluate T-cell exhaustion, a dysfunctional T-cell state that arises during chronic infections, sepsis and cancer, contributing to the progression of these diseases [3]. Loss of T-cell function allows LCMV Cl13 to establish a persistent infection in certain strains of mice. Recent reports have indicated that in certain strains of mice, such as FVB/N [4], NZB [10] and PL/J [41], T cells do not become exhausted and instead lead to CD8^+^ T-cell mediated immunopathology and death. As circulating levels of 3-IPA positively correlate with survival and T-cell exhaustion and 3-IPA supplementation can delay T-cell-mediated death, we assessed whether 3-IPA affects either T-cell proliferation or CD8^+^ T-cell cytotoxicity. Splenocytes from LCMV-infected or naive FVB/N mice were isolated, stimulated with the CD8^+^ epitope NP118 and assessed for proliferation over five days under different concentrations of 3-IPA. We observed a dose-dependent suppression of proliferation of splenocytes from infected FVB/N mice (Figure 4A). Similarly, when proliferation of CD8^+^ T cells was assessed, we observed a dose-dependent suppression of proliferation as well. We observed a similar suppression of proliferation of CD8^+^ T cells from naive animals (Figure 4A). The ability of 3-IPA to suppress basal proliferation in naive animals suggests that 3-IPA may act as a general inhibitor of T-cell proliferation, even in the absence of a specific antigen.

Because CD8^+^ T-cell function is critical for LCMV Cl13-mediated death in susceptible mice, we assessed whether 3-IPA affects the cytotoxic function of these cells. Target cells pulsed with NP118 peptides were labeled with a high concentration of cell trace violet (CTV^hi^), while target cells pulsed with an irrelevant peptide were labeled with a lower concentration of CTV (CTV^lo^). CTV^hi^ and CTV^lo^ cells were mixed 1:1 and incubated with splenocytes from LCMV-infected FVB/N mice (6 days post-infection) at the indicated ratios. Cell killing was assessed by comparing cells positive with 7-amino-actinomycin D (7-AAD) viability dye to those negative for 7-AAD in each CTV population. 7-AAD intercalates between cytosine and guanine bases of the DNA and is a reagent allowing for differentiation of viable from nonviable cells using flow cytometry. Both the intensity and number of 7-AAD positive cells decreased upon an increasing concentration of 3-IPA (Figure 4B).

Taken together, these data indicate that 3-IPA has an immunosuppressive role in these cells when it is added exogenously, reducing CTLs’ ability to kill target cells and proliferate (Figure 4). This is consistent with 3-IPA levels observed in the metabolomics experiments, where the presence of 3-IPA in plasma is correlated with a T-cell exhaustion phenotype that allows for their survival, whereas a dramatic decrease in circulating 3-IPA may result in a reversal of the immunosuppressive activity and contribute to acute activation of CTLs resulting in death.

## 3. Materials and Methods

### 3.1. Mice and Viruses

All mouse strains (i.e., C57Bl6/J, FVB/N, DBA/1J, and DBA/2J) were obtained from the rodent breeding colony at the Scripps Research Institute. DBA/1J and DBA/2J were initially obtained from Jackson Laboratory, Bar Harbor, Maine. All mice were maintained according to strict adherence to standards and protocols approved by the TSRI Institutional Animal Care and Use Committee and AAALAC. Mice were infected intravenously with 2 × 10^6^ focus-forming units of LCMV Cl13. The LCMV Cl13 virus stocks were grown and quantified as previously described [4,10]. Mice were euthanized under isofluorane, and spleens were isolated and subsequently dissociated into a single-cell suspension by incubation with collagenase D (1 mg/mL) and DNaseI (100 µg/mL) in RPMI and processing through a 100 µm nylon mesh.

### 3.2. Proliferation Assay

Splenocytes from mice six days after infection with LCMV Cl13 were suspended at 4.0 × 10^7^ in 200 µL of RPMI without serum and rapidly diluted in 800 µL of cell trace violet (CTV, Invitrogen (Carlsbad, CA, USA) #c34571) diluted at 1:800 (CTVhi) and incubated for 20 min at 37 °C covered from light. Staining was quenched with four milliliters of prewarmed R10 (RPMI with 10% FBS, 1% p/s and 1% L-glutamine) and allowed an extra five minutes at 37 °C. Cells were plated in a round-bottomed 96-well plate, 3-IPA was added at the indicated final concentrations and incubated at 37 °C with 5% CO_2_ for 5 days. Cells were washed once, then resuspended in 50 uL of FACS buffer (2% FBS in PBS) containing 1:200 CD8-PE (Biolegend, San Diego, CA, USA) and CD3-FITC (Biolegend) for 30 min on ice and covered. Cells were washed 2× with 200 µL FACS buffer, suspended with 1 uL of 7-AAD in 400 µL of FACS buffer and acquired by flow cytometry.

### 3.3. CTL Assay

Splenocytes from day 6 post-infection or uninfected FVB/N mice were isolated as in proliferation assay and rapidly diluted in 800 ul of cell trace violet (CTV, Invitrogen #c34571) diluted at 1:800 (CTVhi) or 1:1200 (CTVlo) and incubated for 20 min at 37 °C covered from light. Staining was quenched as above. Cells were then spun at RT 1500 RPM 5 min, aspirated, resuspended in 1 mL with either 2 µg LCMV GP33 peptide for CTV^lo^ (nonspecific) or 2 µg of NP118 for CTV^hi^. Cells were allowed to absorb peptide for 30 min in a 37 °C incubator, then washed with twice with 4 mL R10. CTV^lo^ and CTV^hi^ samples were combined at 1:1 and dispensed as “target” cells in 96-well round-bottomed plates. Effector splenocytes from infected or uninfected FVB/N mice were suspended in IPA3 containing R10 starting at 5290 µM and diluted accordingly. These effector cells were added at effector:target ratios of 50:1, 12:1 and 0:1 (no effectors) for 3 h before adding 1 µL of 7-AAD (#559925 BD) per sample, mixed and equilibrated for 20 min RT covered. Specifically lysed cells were gated based on CTV^hi^ NP118+ gate and subtracted from nonspecific CTV^lo^ GP33+ gate.

### 3.4. Flow Cytometry and Data Analysis

All flow cytometry was acquired on a LSRII using Diva software and analyzed with FlowJo Software. Other data were graphed and analyzed using GraphPad Prism software (v 9.0, San Diego, CA, USA).

### 3.5. Untargeted Metabolomics

Samples were analyzed by three independent liquid chromatography/mass spectrometry (LC/MS) platforms. Metabolites were extracted from plasma by adding four volumes of ice-cold methanol/acetonitrile 1:1. To precipitate plasma proteins, samples underwent three cycles of freeze/thaw (liquid nitrogen for 1 min). Afterwards, samples were incubated at −20 °C for 60 min. Finally, proteins were pelleted by centrifuging samples at 16,000× *g* for 15 min at 4 °C. Supernatants were dried down under vacuum and reconstituted in 100 μL of acetonitrile/water 1:1 prior to LC/MS analysis by reversed-phase (RP) chromatography operated in positive ion mode and hydrophilic liquid interaction chromatography (HILIC) operated in both the positive and negative ion modes. The LC/MS analyses were carried out in a Bruker Impact II quadrupole/time-of-flight (q-ToF) mass spectrometer coupled to a Bruker Elute UHPLC (Bruker, Billerica, MA, USA). Data were acquired over a mass/charge ratio (*m*/*z*) range of 50 to 1000 Da. The electrospray source conditions were as follows: end plate offset, 500 V; dry gas temperature, 200 °C; drying gas, 6 L/min; nebulizer, 1.6 bar; capillary voltage, 3500 V. Sodium formate was used for post-run mass calibration. The same mobile phases were used for both RP and HILIC positive chromatography, consisting of 0.1% formic acid in water (*v*/*v*) as phase A and 0.1% formic acid in acetonitrile (*v*/*v*) as phase B. For HILIC negative, solvents were the same with the addition of 1 mM ammonium formate + 0.1% formic acid. The flow through the column in both cases was 150 μL/min. An ACQUITY BEH C18 column (1.0 by 100 mm, 1.7 μm, Waters Corporation, Milford, MA, USA) was used for the RP analysis and an ACQUITY BEH Amide column (1.0 by 100 mm, 1.7 μm, Waters Corporation) was used for the HILIC (positive and negative) analysis. The gradient for RP chromatography consisted of 99% phase A for 1 min, 1% phase A over 9 min and held at 1% phase A for an additional 3 min. Data were acquired in positive ion mode. The gradient for HILIC consisted of 1% phase A for 1 min, 35% phase A over 13 min, 60% phase A over 3 min and held at 60% phase A for an additional 1 min. Data were acquired in the positive and negative ion modes in independent runs. The injection volume was 2 μL. For identification purposes, putative molecules of interest were fragmented at three different collision energies (10, 20 and 40 eV).

### 3.6. Mass Spectrometry Data Analysis

Raw LC-MS data were converted to the mzXML format using ProteoWizard MS Converter version 3.0.7529 (Palo Alto, CA, USA). The mzXML files were uploaded to XCMS Online for data processing. Peaks were first detected, aligned across samples and integrated. Then, features (a set of integrated peaks with a particular *m*/*z* and retention time) underwent isotope removal and adduct and in-source ion annotation. Data were processed using the following parameter settings: centWave for feature detection (Δ *m*/*z* = 15 parts per million, minimum peak width = 10 s and maximum peak width = 60 s); obiwarp settings for retention time correction (profStep = 0.5); parameters for chromatogram alignment including mzwid = 0.015, minfrac = 0.5 and bw = 5. Lastly, after the statistical analysis, only features with *q* < 0.05 (one-way ANOVA followed by a Kruskal–Wallis nonparametric test) were selected for identification through MS/MS experiments. To identify these metabolites, the resulting MS/MS spectra were matched against the METLIN database [37].

### 3.7. Bioinformatics

Cognitive Prioritization of Metabolites: Dysregulated metabolites were ranked using IBM Watson for Drug Discovery platform (IBM, Armonk, NY, USA). A list of keywords and entities related to T-cell exhaustion, immune response and systemic infection were used as the known set that candidate metabolites were ranked against. The candidate metabolites were inputted and disambiguated into drug or chemical entities, which contained chemical and structural information, common annotations and known synonyms. Medline Abstracts were queried, and the candidate metabolites were given a similarity score and ranked based on similarity to keywords and entities known to be related to temperature control. Results were validated by reranking the metabolites against any random three of the five biological entities used as the know set.

Pathway enrichment analysis: Pathway enrichment was performed using the Wikipathways database and a custom script using each metabolite’s fold change.

## 4. Conclusions

Metabolomics was used to examine T-cell exhaustion and immunosuppression, revealing the unique role of 3-IPA, a microbial-derived metabolite [1]. To accomplish this, two genetically related mouse strains were infected with LCMV Cl13, producing disparate outcomes driven by T-cell responses. These models showed a metabolic response to viral infection with over 70 metabolites altered over the course of the infection, most of them in mice with a fatal outcome. Among them, purines, energy metabolites and amino acids were significantly dysregulated between mice that survived and died. Using cognitive computing analysis combined with fold change and statistical significance, the 70 metabolites were prioritized with 3-IPA, a tryptophan-derived metabolite synthesized by gut microbiota, identified as a high-priority candidate for testing of immune modulation. Previous efforts have shown 3-IPA to have multiple unique physiological characteristics [42] and is essential for mice survival through the negative immune regulation including targeted cell killing and proliferation. 3-IPA was added to the drinking water of the mice with results showing that 3-IPA allowed the mice to survive longer against fatal LCMV Cl13 infection. This negative immune-modulation effect might be of interest for the modulation of CTL responses in events such as sepsis, autoimmune diseases, type I diabetes, organ transplantation or even COVID-19. Moreover, 3-IPA’s bacterial origin raises the possibility of targeting the microbiome to enhance CTL responses in diseases such as cancer and chronic infection.

## Figures and Tables

**Table 1:** Metabolic feature analysis performed in the three untargeted metabolomics platforms used for this study prior to LCMV infection (day 0). The percentage of changed features was calculated in respect to the total number of measured features for each analytical platform. |fc| > 2 represents features with a fold change between strains that was greater than 2 (or lower than 0.5). |fc| > 5 represents features with a fold change between strains greater than 5 (or lower than 0.2). Only features with an intensity over 5000 counts were considered for the analysis. RPpos: reversed-phase positive ion mode; HILICpos: hydrophilic interaction liquid chromatography positive ion mode; HILICneg: hydrophilic interaction liquid chromatography negative ion mode.

Changed Features (% of Total)	RPpos |fc| > 2	RPpos |fc| > 5	HILICpos |fc| > 2	HILICpos |fc| > 5	HILICneg |fc| > 2	HILICneg |fc| > 5
F vs. B at day 0 (non-infected)	24.2	7.4	21.5	5.8	14.5	2.4
DBA1 vs. DBA2 at day 0 (non-infected)	8.7	0.8	13.8	2.5	11.0	1.5

## Data Availability

XCMS raw data were deposited into the XCMS Online Public repository.

## Supplementary Materials

The following supporting information can be downloaded at: https://www.mdpi.com/article/10.3390/metabo12070645/s1, Table S1: Summary of dysregulated metabolites in the two mice models during LCMV infection; Table S2: Cognitive prioritization of metabolites using the IBM Watson for Drug Discovery plastform.
